# In Silico Analysis of the Dual Role of Tumor Microenvironment on Colon Cancer Subtypes

**DOI:** 10.1177/11769351261431245

**Published:** 2026-03-26

**Authors:** Christianah Kehinde, Michelle Livesey, Yeuko Manganyi, Hocine Bendou

**Affiliations:** 1 Computational Biology Division, Faculty of Health Sciences, Integrative Biomedical Sciences, University of Cape Town, South Africa; 2 Department of Pathology, Faculty of Health Sciences, Institute of Infectious Diseases and Molecular Medicine, University of Cape Town, South Africa

**Keywords:** colon cancer, heterogeneity, subtyping, tumor microenvironment, targeted therapy

## Abstract

**Background:**

Colon cancer is a highly heterogeneous disease, marked by substantial intra- and inter-tumor variability. Investigating transcriptomic profiles can offer deeper insight into this heterogeneity. However, most genome-transcriptome studies on colon cancer have primarily focused on examining primary tumors and matched normal tissues, often neglecting the multi-stage disease progression.

**Objective:**

To establish unique molecular colon subtypes based on the progression in transcriptomic profiles. Additionally, to investigate the implicated factors, such as mutations and the tumor microenvironment (TME), that affect colon cancer progression and their implications for therapy.

**Methods:**

RNA-sequencing data from The Cancer Genome Atlas Colon Cancer (TCGA-COAD) cohort were obtained from the UCSC Xena database, including 47 early and 39 late-stage tumor samples. Heterogeneity was exposed by tracking cancer progression through the multi-stages of cancer development. Hierarchical clustering revealed colon subtypes with varying progression, and differentially expressed genes (DEGs) were identified between these subtypes. The DEGs were subjected to Recursive Feature Elimination and mutational analyses to reveal driver genes. The TME and biological pathways were analyzed. The study was validated with an independent GEO dataset.

**Results:**

Two novel colon subtypes were identified. Significant enrichment pathways and varied mutations in cancer driver genes were found in both subtypes. Interestingly, concurrent downregulation of oncogenes and tumor suppressor genes was observed in one of the subtypes, suggesting a link to the dual functionality of CD4 and CD8 T-cells in the TME.

**Conclusion:**

Overall, our study demonstrates a complex relationship between TME and gene expression of driver genes. The presence of immune cell fractions with dual functions suggests a balanced early-to-late-stage progression. The findings provide insights into the disease progression that potentially contribute to the development of targeted therapies.

## Introduction

Colon cancer is marked by the uncontrolled growth of malignant cells in the large intestine. It frequently develops from adenomatous polyps on the colon’s inner lining.^1,2^ According to the Global Cancer Statistics 2020, colorectal cancer is the second leading cause of cancer-related mortality, but ranks third in terms of incidence.^3,4^ The incidence rates of colon cancer vary significantly by region, with the highest rates observed in North America, Europe, and Australia/New Zealand.^3,5^ The disease develops through a tumor-initiating event involving genetic or epigenetic alterations that activate oncogenes or inactivate tumor suppressor genes (TSGs), followed by clonal expansion and subsequent metastasis.^6,7^ The TNM Classification system, based on tumor invasion (T), lymph node involvement (N), and metastases (M), is the primary prognostic tool, with 5-year survival rates of 94% for stage 1, 82% for stage 2, 67% for stage 3, and 11% for stage 4 in the disease progression.^7^ Treatment includes surgery for early stages, with adjuvant chemotherapy for stage 3 and high-risk stage 2 tumors. For stage 4, options include metastasis resection, palliative chemotherapy, and radiotherapy for symptom management.^6,8,9^ Despite advances in diagnostic and therapeutic approaches, survival remains highly variable even within the same TNM stage.^6,9^ Although the TNM system has prognostic importance, it is limited in its ability to accurately identify high-risk patients for both preoperative and pathological staging. This issue is relevant across all stages of colorectal cancer, especially in the locally advanced stage.^10^

Over the past decade, colon cancer has been increasingly recognized as a highly heterogeneous disease, characterized by significant intra-tumor and inter-tumor heterogeneity. This heterogeneity is a critical factor underlying the wide variation in oncological outcomes and prognostic trajectories observed among patients.^9,11^ Colon cancer, being a highly heterogeneous disease, involves DNA repair defects, DNA methylation, chromosome instability, and TME, as well as other molecular pathogeneses in the multi-stage cancer development that influence prognosis and recurrence risk in colon cancer.^12-15^ Recent reports have indicated that the TME influences colon cancer heterogeneity because of its dynamic interactions between immune cells and tumor cells.^13,16^

Diverse response to treatment in patients at different stages has been linked to Inter-tumor heterogeneity caused by mutations and differential gene expression across colon cancer from various patients.^11,17^ Therefore, the gene expression patterns approach provides a more comprehensive view of inter-tumor heterogeneity and plays a crucial role in identifying diagnostic and predictive markers.^11^ The approach considers heterogeneity influenced by the composition and impact of the TME, and the differential activity of cell signaling pathways in cancer cells that are not necessarily regulated solely by mutations.^11^ The approach could be further implemented in the primary concern of standardized staging and treatment protocol, which fails to predict the appropriate stage for the whole cohort of colon cancer patients to receive adjuvant therapy, and reasons for poor prognosis across the stages.^18^ This highlights the importance of gene expression analysis across the multi-stage disease progression.

The heterogeneity is further evident in the possible dual behavioral functions of immune^19-21^ cells, acting both as anti-tumorigenic and pro-tumorigenic agents in the TME.^22^ The immune cells act either as inhibitors of tumors (anti-tumor immunity) or as promoters of tumors (pro-tumor immunity), a “double-edged sword” within the TME that contributes to the observed heterogeneity.^22^ Due to the high heterogeneity of the disease, no gene expression characteristics are reliable for prognostic stratification in clinical settings.^19-21^

Recent studies underscore the potential of transcriptomic analysis to uncover the heterogeneity of colon cancer.^23,24^ Transcriptomics links molecular mechanisms to cellular activities, with RNA-seq enabling precise quantification of transcriptional outputs at single-cell and bulk levels.^24,25^ However, most genome-scale research is case-control studies, often neglecting the multi-stage progression of cancer from early to advanced stages.^24,26^ Examining transcriptomic changes across disease progression offers opportunities to identify novel subtypes, as RNA-seq variation during cancer development remains underexplored. According to the consensus molecular subtypes,^27^ 4 molecular subtypes were previously identified and reported: CMS1 (MSI-immune), CMS2 (canonical), CMS3 (metabolic), and CMS4 (mesenchymal), based on aggregated gene expression; however, these classifications did not account for stage-specific RNA-seq differences. These subtypes (CMS1-CMS4) highlight distinct biological characteristics and potential therapeutic responses. Moreover, the CMS classification was mainly derived from early-stage samples, with 92% of samples representing early-stage tumors at diagnosis.^28^ It is therefore imperative to go beyond the primary tumor and incorporate studies that provide insight into how tumor characteristics evolve throughout the metastatic process.

Additionally, a potential limitation to consider is that the CMS Subtypes classification can differ across sampling sites.^29^ This intratumor heterogeneity might undermine the reliability of the CMS classification, and variations may exist between the tumor center and the invasive front, as well as between primary tumors and their metastases.^28,29^ Our new subtypes aim to complement this existing framework by providing a more granular understanding of colon cancer progression based on specific RNA-seq expression patterns. In the era of personalized medicine, identifying novel subtypes based on cancer stage progression is crucial for recognizing inter-tumor heterogeneity and advancing targeted therapy development.

Tracking the progression of colon cancer provides valuable insights into the dynamic changes in the cellular transcriptome and deepens our understanding of the molecular mechanisms underlying carcinogenesis. This approach enables an efficient and thorough depiction of the gene expression patterns that vary over time across different conditions. Examining variations among identified subtypes enhances understanding of the characteristics and extent of heterogeneity in colon cancer. Furthermore, this knowledge enables the discovery of biomarkers and driver genes, thereby streamlining the screening process and improving prognosis.

In this study, a recently developed normalization method will be employed to capture the heterogeneity of colon cancer cells. By analyzing gene expression through RNA-seq, this method identifies molecular variations in tumor progression from early to late stages.^30,31^ Hierarchical clustering will subsequently be applied to visualize clusters with distinct progression patterns, enabling the sub-classification of heterogeneous colon cancer into newly identified molecular subtypes. The research also examines differentially expressed genes (DEGs), mutational variation, the TME, and pathways across subtypes. Additionally, analysis of DEGs to identify optimal gene subsets within cancer clusters was explored to refine subtype characterization. Previously established frameworks to stratify colorectal cancer, including microsatellite instability (MSI),^32^ the CpG island methylator phenotype,^33^ and transcriptome-based consensus molecular subtypes (CMS),^27^ capture unique genomic and immunological characteristics of the disease. In this study, we built on previous research by identifying 2 transcriptome-derived subtypes, labeled L and S, tailored to the specific cohort we analyzed. While our dataset excluded corresponding methylation or MSI profiles, these subtypes present an additional population-level insight that may enhance existing classification systems.

## Methods

### Dataset Collection and Preprocessing

**Figure 1.**
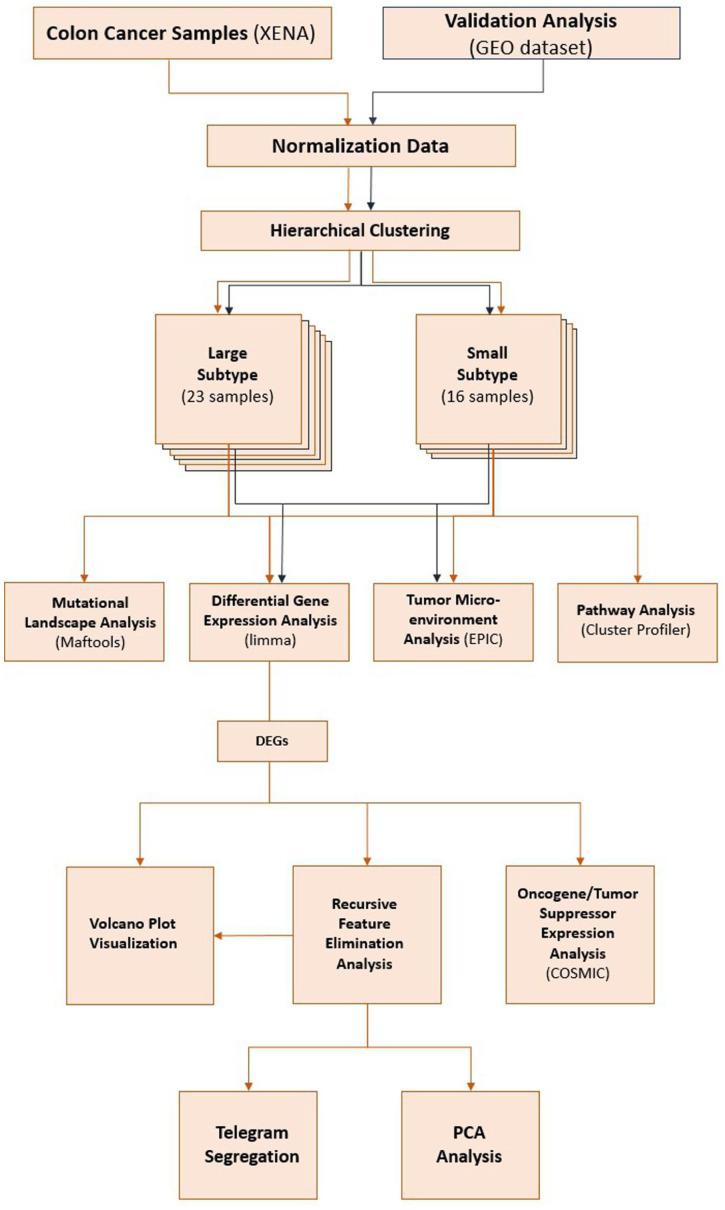
Study workflow. Steps used to analyze the dual role of the tumor microenvironment on newly identified subtypes of colon cancer. The steps comprise data extraction, normalization, differential gene expression, TME analysis, mutational analysis, pathway analysis, and validation.

RNA-Seq data from the GDC TCGA-COAD project were obtained from the UCSC Xena database.^34^ The dataset included log2 (count + 1) normalized expression data, phenotypic information, survival data, and somatic mutation profiles. Patient samples without matched phenotypic and gene expression profiles were discarded. To further reduce the influence of comorbidities on tumor progression, patients older than 75 years were excluded. We however acknowledge that this might affect the generalization of our findings in older patients. Additional selection criteria included a primary diagnosis of adenocarcinoma and the use of primary tumor samples, ensuring a specific focus on colon cancer cases. The final dataset comprised 47 early-stage and 39 late-stage samples. Gene expression profiles were converted to unnormalized count-based (count + 1) values and structured into a genomic matrix, with samples organized as columns and 60 661 Ensembl gene (ENSG) IDs as rows.^35^ From this dataset, 19 569 ENSG identifiers annotated as protein-coding genes were extracted using Ensembl BioMart (GRCh38.p14, Ensembl 113, April 2024), resulting in the exclusion of 67.8% of non-coding genes.^36^ The study’s workflow is depicted in Figure 1.

### Identification of Colon Cancer Subtypes

To identify molecular subtypes of colon cancer, a tracking cancer progression method was applied.^31^ This method derives the expression matrices E (early) and A (advanced) from early- and late-stage samples, respectively. Initially, “m_*i*_” is calculated as the average expression of each gene across early-stage samples, as shown in equation (1), to account for early-stage patient profiles that differ from those in late-stage samples. Matrix L, demonstrated in equation (2), is then calculated as the quotient of late-stage and m_*i*_, revealing accumulated molecular changes that occur throughout the multi-stages of cancer progression. The technique has demonstrated robust segregation of cancer samples within and across cancer types, based on the dynamics of gene expression profiles.^31^ It has also been successfully applied to identify cancer subtypes with distinct prognoses in kidney renal clear cell carcinoma with different prognoses.^30^

(1) $$m_{i}=\frac{1}{r}\overset{r}{\underset{k=1}{\sum }}E_{i,k}$$

(2) $$L_{i}=ln\left(\frac{A}{m_{i}}\right)$$

In this study, the extracted protein-coding genes were normalized using the Livesey et al^31^ method to capture molecular differences associated with tumor progression. Hierarchical clustering (HC) was then employed to visualize and identify colon cancer subtypes. Clustering was performed using the cosine distance between gene expression profiles, with Ward’s method applied for agglomeration.^37^ The optimal number of subtypes (*k*) was determined using the *find_k* function from the dendextend R package (version 1.18.1). The value of *k* was computed based on maximal average silhouette widths, which assesses cluster cohesion and separation.^38^ Finally, the dendrogram was divided into *k* groups, and samples were assigned to the corresponding colon cancer subtypes.

### Differential Gene Expression Analysis

Differential gene expression analysis was performed on 19 569 protein-coding genes across 39 late-stage samples to identify DEGs between the identified colon cancer subtypes. The Limma package in R (version 3.60.6)^39^ was employed for this purpose, utilizing an empirical Bayesian approach to assess and detect significant changes in gene expression patterns between subtypes. The L subtype was used as the reference, while the S subtype was used as a test in the contrast matrix. DEGs were defined based on a Benjamini–Hochberg (BH) adjusted *P*-value threshold of <.05 and a log2-fold change (LFC) of ⩾1.5 or ⩽−1.5, ensuring robust statistical significance and biological relevance. The decideTests tool^40^ was used to identify and extract genes that distinguish the upregulated and downregulated expressed genes. Results were visualized using the EnhancedVolcano R package (version 1.22.0),^41^ which generated volcano plots that provide an intuitive representation of gene expression changes and their statistical significance across subtypes.

### Identification and Selection of Recursive Feature Elimination (RFE) Genes in Colon Subtypes

To identify the most predictive genes associated with colon cancer subtypes, the scikit-learn Python package^42^ was utilized. The RFE algorithm, implemented with a linear kernel support vector machine (SVM), was applied to iteratively rank and select significant genes using DEGs as input. Through iterative feature elimination, less significant genes were systematically removed, and a Repeated Stratified K-Fold cross-validation approach with 7 splits and 7 repeats was employed to ensure robustly selected features. The final gene subset, selected using a linear SVM with a regularization parameter (C) set to 5, achieved the highest classification accuracy. Principal component analysis (PCA) was performed on the final gene subset using the FactoMineR (version 2.11)^43^ and Factoextra (version 1.0.7)^44^ R packages to explore variance and clustering patterns. Normalized gene expression profiles of each RFE selected gene were visualized using box plots, highlighting their distributions across colon cancer subtypes, and revealing subtype-specific expression patterns.

### Differential miRNA Expression and Target Gene Analysis

Colon adenocarcinoma miRNA expression data were obtained from The Cancer Genome Atlas using the Bioconductor package TCGAbiolinks. Raw read counts from the miRNA expression quantification files, processed using the BCGSC miRNA profiling pipeline, were downloaded and merged into a numerical count matrix. Subtype S samples (n = 16) and Subtype L samples (n = 23) were compared to early-stage samples (n = 47) in a differential expression analysis using the edgeR-limma-voom pipeline. Low-expressed miRNAs were filtered out using edgeR filterByExpr() (with the group design and min.count = 10), which removes miRNAs with insufficient expression (CPM/counts) across an appropriate number of samples given the library sizes and experimental design. Library sizes were normalized using the trimmed mean of M-values (TMM) method via edgeR calcNormFactors(method = “TMM”). Differentially expressed miRNAs were identified using the limma-voom method by applying limma voom() to obtain log2-CPM values with precision weights, followed by linear modeling (lmFit()) and empirical Bayes moderation (eBayes()), and BH adjusted *P*-values and LFC were reported. Significant miRNAs were defined as those with an adjusted *P*-value ⩽ .05 and an absolute LFC ⩾ 1. Mature miRNA IDs corresponding to precursor IDs were retrieved using Bioconductor’s miRbaseConverter package, which was used to identify validated miRNA-target interactions involving TSGs and oncogenes by querying the miRTarBase and TarBase databases using the multiMiR package (validated interactions retrieved with multiMiR get_multimir(table = “validated”)). Only experimentally validated interactions categorized as support_type == “Functional MTI” were retained as a high-confidence subset of functionally supported validated MTIs (per the evidence annotation returned by multiMiR). Statistical analyses and visualizations were performed using R software (version 4.3.3).

### Pathways Analysis

To elucidate the biological roles and functions of genes within the identified subtypes, the DEGs were subjected to Gene Ontology (GO) and Kyoto Encyclopedia of Genes and Genomes (KEGG) pathway analyses. These analyses were conducted using the enrichGo and enrichKEGG functions from the clusterProfiler R package (version 4.12.6),^45^ with a significant threshold of *P*-value < .05. GO analysis categorizes gene functions into 3 domains: Biological Process (BP), Molecular Function (MF), and Cellular Component (CC), providing a comprehensive functional annotation framework.^46^

Further enrichment pathway analysis was conducted using Reactome, a curated library of human biological pathways, and reactions.^47^ The list of DEGs was submitted to the Reactome web program (https://reactome.org) for analysis. The over-representation method, with a default false discovery rate (FDR), was employed. The analysis used Reactome database release 92 and Reactome pathway browser version 3.7, accessed on May 15, 2025.

### Mutational Landscape in Colon Subtypes

The mutational and somatic landscape of the colon cancer subtypes was analyzed and visualized using the Maftools R Bioconductor package (version 2.20.0).^48^ This tool processes Mutation Annotation Format (MAF) files to extract detailed mutational data for each subtype. Oncoplots were generated to display the top mutated genes, mutation frequencies, and differences in mutational patterns across the subtypes. Additionally, the oncoplots provided insights into the variant classification and types, offering a comprehensive overview of the mutational characteristics specific to each colon cancer subtype.

### Tumor Microenvironment in Colon Subtypes

The Estimating the Proportions of Immune and Cancer Cells method (EPIC)^49^ was employed to quantify the distribution of immune cell fractions within the TME of the identified colon cancer subtypes. EPIC is recognized for its high precision in estimating immune-cell fraction compared to other tools, offering a reliable approach for analyzing complex tumor ecosystems. To account for the substantial variability in cancer cell types among patients, EPIC incorporates an algorithm that adjusts for unidentified and potentially diverse cell populations within each subtype. The method models bulk RNA-Seq data to predict cell-type fractions in mixed RNA-Seq samples, utilizing reference gene expression patterns of major immune and non-malignant cell types. We also utilized the xCell tool^38,39^ alongside the EPIC tool to estimate the immune cell fractions. The normalized gene expression data were entered into the xCell web tool (https://xcell.ucsf.edu/). This analysis assigns the proportions of each cell fraction to every sample in the input data. EPIC utilizes constrained regression to estimate absolute proportions accurately and is tailored for tumor samples that exhibit clearly defined immune and stromal characteristics.^50^ In contrast, xCell generates enrichment scores rather than absolute values, making it attuned to pathway-level signals but possibly less precise in quantification.^51,52^ Using both tools introduces methodological variation, and differences in results may stem from varying definitions of cell types, marker specificity, or assumptions regarding tumor purity.^50-52^ This approach provides a comprehensive characterization of the TME, facilitating insights into the immune composition and heterogeneity across the colon cancer subtypes.

Spearman rank correlations were computed using the SciPy spearmanr function between EPIC-inferred CD4 and CD8 T-cell fractions and the expression of oncogenes and TSGs from DEGs across matched samples. Two-sided *P*-values were corrected for multiple testing using the BH FDR procedure. In addition to single-gene analyses, gene-set scores, were calculated using the Scipy zscore function. These scores were defined as the mean gene-wise z-scored expression across samples for both the oncogenes and the TSGs. The correlation between these scores and the CD4/CD8 fractions was then determined. To account for potential confounding by bulk tissue composition, we used the EPIC-estimated otherCells and performed purity-adjusted analyses. Specifically, we fit linear models of the form *score ~ subtype + otherCells* for oncogene and TSG gene-set scores, and *expression ~ subtype + otherCells* for the RFE-selected genes. *P*-values were corrected using the BH procedure.

### Validation

The proposed framework was validated using the independent dataset GSE17538, obtained from the Gene Expression Omnibus (GEO) database.^53^ The sequencing data were generated using the GPL570 platform ([HG-U133_Plus_2] Affymetrix Human Genome U133 Plus 2.0 Array). Probe identifiers were converted to gene symbols using the BioMart platform^36^ to ensure consistency with the primary dataset. Because GSE17538 is microarray-based GPL570 platform, not all RNA-seq defined Ensembl IDs were represented on the platform. Therefore, external validation was performed using the overlap subset (6 genes) available in GSE17538 from the 16 RFE genes (Supplemental S1 Table). To account for cross-platform variability between RNA-seq and microarray measurements, gene expression values were standardized within each dataset (gene-wise z-score across samples) prior to model training/testing. A linear SVM classifier (linear kernel; *C* = 5) trained on the UCSC Xena dataset using the overlap genes was then applied to GSE17538 without retraining. Model performance in the validation dataset was assessed using ROC AUC and classification metrics (accuracy, F1-score, confusion matrix), and subtype separation in the 6-gene space was additionally quantified by Python sklearn silhouette width, treating the external subtype labels as cluster assignments. For validation, 45 late-stage and 18 early-stage samples were selected based on the preprocessing selection criteria outlined in Section 2.1, enabling a robust comparison and evaluation of the framework’s performance.

## Results

### Identified Colon Cancer Subtypes

**Figure 2.**
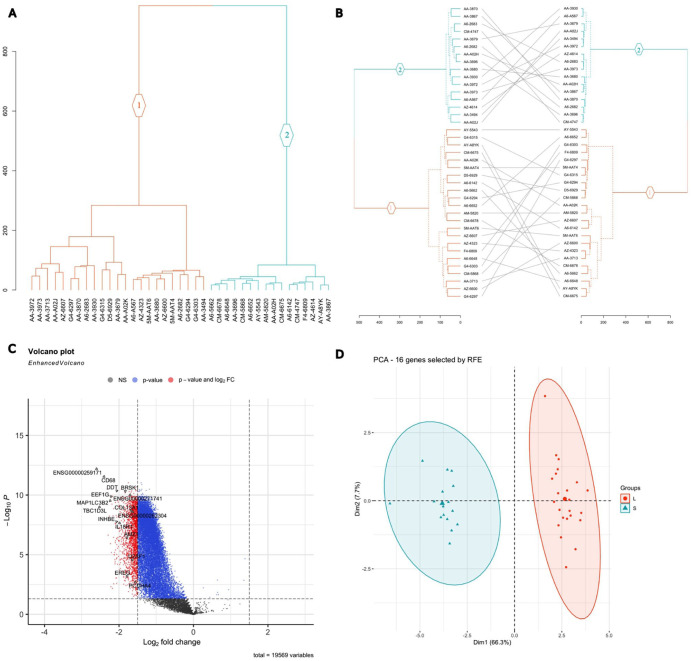
Colon cancer subtyping. (A) Hierarchical clustering dendrogram of colon cancer samples on the x-axis. Two subtypes, L (orange branches) and S (blue branches), were identified using hierarchical clustering of the 19 569 protein-coding gene expression profiles from 39 colon cancer samples. (B) Tanglegram comparing hierarchical clustering outcomes. The contrast of the HC of all 19 569 normalized protein-coding genes (left) and HC with the normalized gene expression of the RFE 16-gene subset (right). Samples are displayed along the y-axis, and the lines connecting the 2 dendrograms indicate the correspondence between the samples in the 2 clusterings. (C) Volcano plot of the 19 569 normalized protein-coding genes in the 39 late-stage samples. The dashed horizontal line indicates a statistical significance threshold (adjusted *P*-value ⩽ .05). Two vertical dashed lines indicate the thresholds for log2-fold change ⩾ 1.5 and ⩽−1.5. The color points indicate whether the genes are non-significant (gray), have adjusted *P*-values ⩽ .05 (blue), or have significant log2-fold changes and adjusted *P*-values (red). The plot also labels the selected RFE gene subset. (D) Principal Component Analysis of the normalized gene expression profiles of the RFE 16-gene subset. The analysis highlights stratification of colon cancer samples into the L (orange ellipse) and S (blue ellipse) subtypes, with a clear separation in the 2D PCA space.

Applying the method of Livesey et al^8^ on the extracted protein-coding genes of the 39 late-stage samples, with the early-stage samples used as a reference, resulted in normalized counts that revealed differences between stage groups in gene expression in colon cancer samples. Hierarchical clustering revealed distinct early- to late-stage patterns of gene expression. The HC dendrogram (Figure 2A) identified 2 distinct colon cancer subtypes, a larger subtype comprising 23 samples (orange branches) and a smaller subtype of 16 samples (blue branches), hereafter referred to as the L (Large) and S (Small) subtypes, respectively.

This classification was validated using the independent dataset GSE17538, which similarly identified 2 clusters: a large cluster of 31 samples and a small cluster with 14 samples (Supplemental S1 Figure). The consistency in the number of clusters and the Large and Small identified clusters between the discovery (TCGA-COAD) and validation (GSE17538) datasets underscores a possible existence of stage difference heterogeneity in colon cancer and the potential relevance of understanding it.

### Differentially Expressed Genes in Colon Cancer

Analysis of differential gene expression revealed significant differences between the 2 subtypes (S and L), with L being used as the reference subtype. A total of 1855 DEGs were identified (Figure 2C), based on a threshold of LFC ⩾ 1.5 or ⩽−1.5, and an adjusted *P*-value < .05. Most of the 1855 DEGs were downregulated in subtype S compared to the reference L. This suggests that these DEGs underwent greater normalized gene expression downregulation in subtype S than in subtype L. This distinct expression pattern highlights substantial molecular differences between the 2 subtypes, underscoring the heterogeneity in gene expression between the subtypes and offering new insights into their underlying molecular characteristics.

### Identified Classification Genes in Colon Subtypes

RFE analysis identified a novel subset of genes associated with enhanced accuracy in categorizing colon cancer patients into the L and S subtypes. From the 1855 DEGs, a refined subset of 16 genes was selected as optimal predictive markers for distinguishing between the S and L subtypes. This 16-gene subset achieved a performance classification score of 0.98, demonstrating high reliability (Supplemental S1 Table).

To validate the clustering capability of the 16-gene subset, hierarchical clustering was carried out using the gene expression profiles of these genes. The resulting clustering was compared to the initial clustering based on all protein-coding genes, with the relationship between the 2 clustering outcomes visualized using a tanglegram, which shows the 2 dendrograms side-by-side (Figure 2B). The tanglegram showed that all 39 samples were consistently classified into the same subtypes (L and S) using the reduced 16-gene set, confirming the robustness of this set for subtype classification.

Using a Principal Component Analysis model, the variation in the gene expression profiles of the RFE 16-gene subset between L and S was assessed. As shown in Figure 2D, dimension 1 accounts for 66.3% of the overall variance, while dimension 2 accounts for 7.7% of the variance. The PCA plot illustrates a clear segregation between the 2 subtypes, with samples from L (replicates with an orange ellipse) and S (blue ellipse) distinctly separated in the 2-dimensional (2D) space.

**Figure 3.**
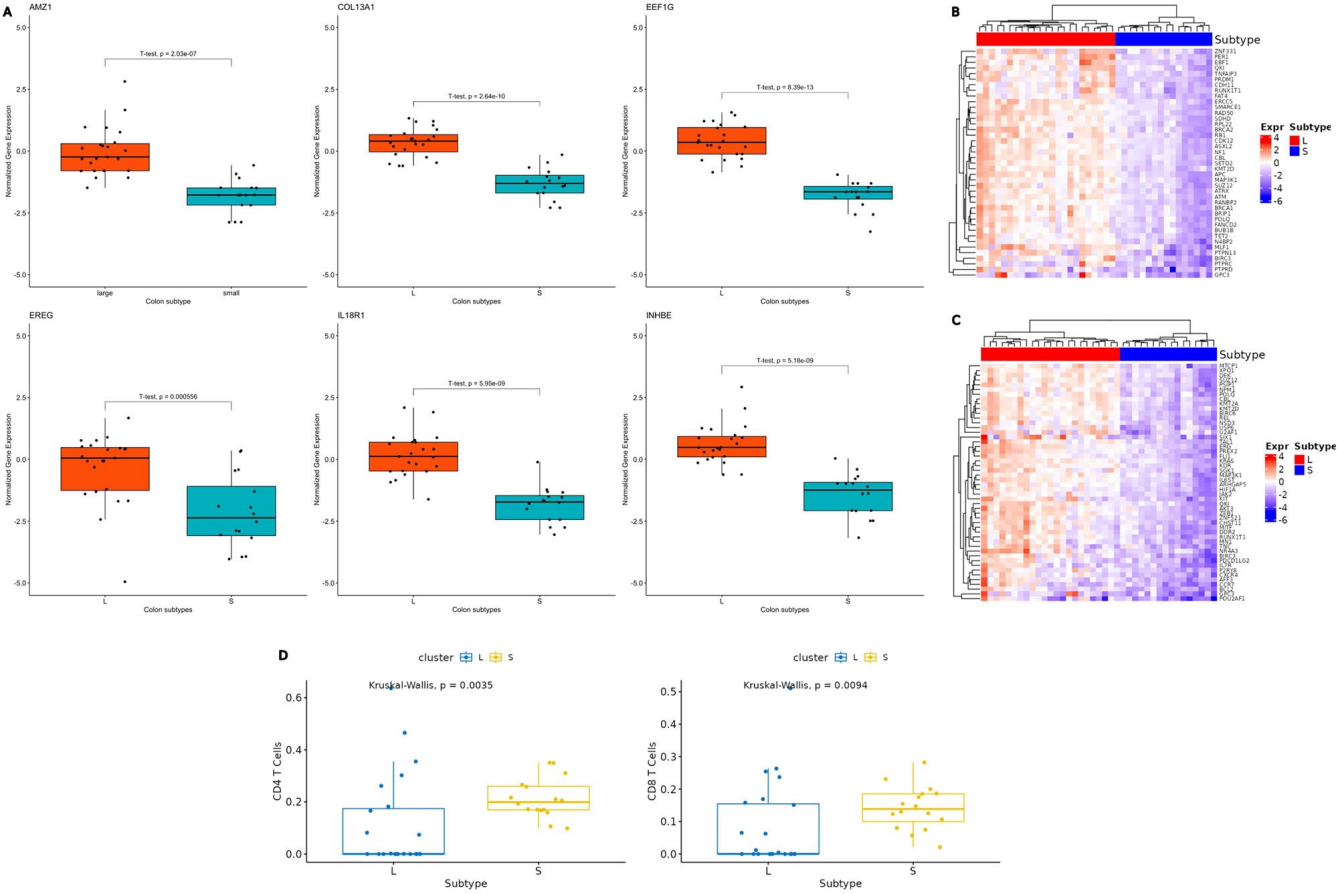
Expression of the colon cancer subtypes. (A) Boxplots of 6 of the RFE 16-gene subset. Boxplots depict the normalized gene expression profiles (*y*-axis) of genes AMZ1, COL13A1, EEF1G, EREG, IL18R1 and INHBE in colon cancer samples classified into the L and S subtypes (*x*-axis) from the Xena dataset. These 6 genes were also identified in the independent GSE17538 dataset and exhibited consistent gene expression patterns across both datasets (Supplemental S2 Figure). (B) A heatmap representation of TSGs and (C) oncogenes. The rows represent gene expression, and the columns represent colon cancer samples categorized into L and S subtypes (color bar at the top). Key abbreviation: Expr = Gene Expression. (D) Boxplots of immune cell fractions for CD4 and CD8 T-cells (y-axis) across subtypes L and S (x-axis). A comparable pattern of immune cell expression was observed in the GEO dataset analysis (Supplemental S3 Figure), further validating the findings.

The expression patterns of the RFE 16-gene subset were evaluated between L and S using boxplots. All 16 genes demonstrated statistically significant differences between the 2 subtypes (T-Test, *P* ⩽ .0015). Validation of the 16-gene subset was performed using the independent GSE17538 dataset, which encompassed 6 of the 16 genes: *AMZ1*, *COL13A1*, *EEF1G*, *EREG*, *IL18R1*, and *INHBE*. The overlap subset showed strong discriminatory performance in GSE17538, ROC AUC of 1.00, with high classification accuracy of 0.95 and F1-score of 0.93 (confusion matrix = [[29,2], [0,14]]). Treating the external labels as cluster assignments, silhouette analysis further supported separation in the 6-gene expression space (mean silhouette width = 0.471; subtype *S* = 0.434; subtype *L* = 0.488). Boxplots of the 6 genes showed statistically significant differences between the L and S subtypes in both the Xena (*T*-test, *P* ⩽ .000556; Figure 3A) and the GSE17538 datasets (*T*-test, *P* ⩽ .00229; Supplemental S2 Figure). The gene expression patterns were consistent across the datasets, with all 6 genes exhibiting downregulation in the S subtype.

### Role of Tumor Suppressor Genes and Oncogenes

In addition, the DEGs were filtered to identify TSGs and oncogenes based on the Cancer Gene Census from the COSMIC database.^54^ The analysis revealed 82 such genes amongst the DEG list, with roughly similar proportions of TSGs and oncogenes. To further investigate their involvement in tumor progression, we examined their normalized gene expression levels. Interestingly, most of the TSGs and oncogenes from the DEGs were downregulated in the S subtype compared to the L subtype (Figure 3B and C).

### Differential miRNA Expression and Target Gene Analysis

**Figure 4.**
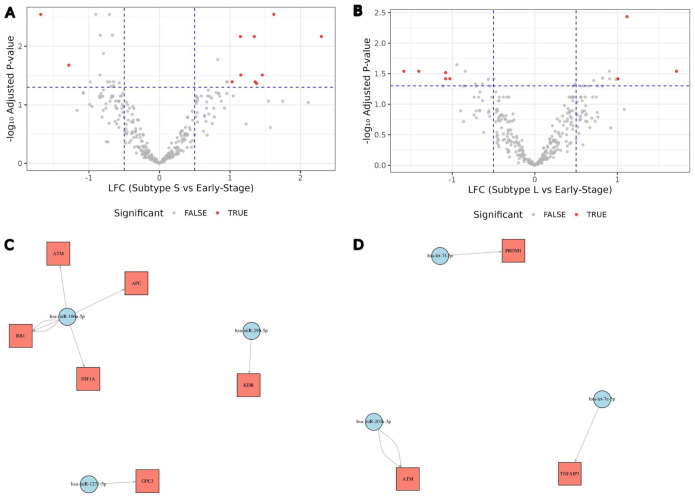
Differential miRNA expression and validation of miRNA-target interaction involving TSGs and oncogenes. (A) Volcano plot illustrating the differential miRNA expression between subtypes S and early-stage. Significant miRNAs are shown in red. (B) Volcano plot illustrating the differential miRNA expression between subtypes L and early-stage. Significant miRNAs are shown in red. (C and D) Target-gene interaction networks of subtypes S and L show literature-curated, experimentally validated MTIs retrieved via multiMiR, restricted to Functional MTI support type; edges indicate previously reported functional evidence and were not experimentally validated in this study.

Differential miRNA expression analysis between subtype S and early-stage samples identified 9 differentially expressed miRNAs (Figure 4A). Validated experimental interactions were found between 3 mature miRNAs, hsa-miR-106a-5p, hsa-miR-296-5p and hsa-miR-1271-5p, and TSGs and oncogenes (Figure 4C). These MTIs were retrieved from multiMiR, and we retained only interactions annotated as Functional MTI to focus on the most stringent evidence category. Interestingly, all the above miRNAs were upregulated in subtype S. This pattern is consistent with the possibility of increased post-transcriptional repression of some targets in subtype S. However, the MTI evidence is derived from curated external studies and does not constitute direct confirmation in our cohort. Additionally, 3 differentially expressed mature miRNAs were also reported between subtype L and early-stage samples: hsa-let-7f-5p, hsa-miR-203a-3p and hsa-let-7e-5p. However, their expression is not consistent and is either upregulated or downregulated, also targeting fewer TSGs and oncogenes (Figure 4B and D).

### Enrichment Pathways Analysis

**Figure 5.**
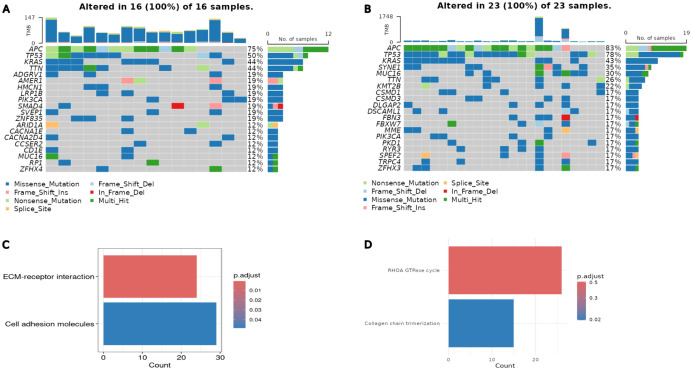
Gene mutations and cellular pathways. (A) Oncoplot of the mutational landscapes of colon subtype S and (B) subtype L. Columns represent individual patient samples, while rows represent genes. The barplot at the top displays the mutation frequency per sample, and the barplot on the right indicates the mutation frequency for each gene. Colored squares indicate mutated genes, while gray squares indicate the non-mutated genes. Multi_Hit = genes that underwent multiple mutations in a single sample. (C) KEGG pathway enrichment analysis. KEGG enrichment analysis of the 1855 DEGs was performed using the clusterProfiler R package, identifying 2 significantly enriched pathways: ECM-receptor interaction and Cell adhesion molecules. (D) Reactome pathway enrichment analysis of DEGs. The top 2 statistically significant pathways are shown, and their colors are shown by the *P*-values.

KEGG pathway analysis of the DEGs revealed 2 significantly enriched pathways: the extracellular matrix (ECM)-receptor interaction and cell adhesion molecules (Figure 5C). The estimated *P*-values for ECM-receptor and cell adhesion molecules are 1.671 × 10^−6^ and 3.044 × 10^−4^, respectively, and the adjusted *P*-values are 5.3 × 10^−4^ and 4.84 × 10^−2^, respectively. This was corroborated by the Reactome enrichment pathway analysis, which highlighted 2 key, functionally linked pathways: Collagen chain trimerization and the RHOA GTPase cycle (Figure 5D). The estimated *P*-values for collagen chain trimerization and the RHOA GTPase cycle on Reactome are 6.52 × 10^−6^ and 6.942 × 10^^−4^, respectively, and the false discovery rates are 1.3 × 10^^−2^ and 5.01 × 10^^−1^, respectively. All values are significant, except for the FDR for the RHOA GTPase cycle. Gene Ontology (GO) pathway enrichment analysis was conducted on the 1855 DEGs using the clusterProfiler R package. The most significantly enriched pathways included the collagen-containing extracellular matrix, centrioles, extracellular matrix cellular constituents, and cell-substrate adhesion, all of which are implicated in colon cancer (Supplemental S4 Figure).^14,36,54,55^

### Mutational Profile Analysis of Colon Subtypes

The mutational landscapes of the identified subtypes were analyzed using the maftools R package and its *oncoplot* function. This analysis revealed significant differences in mutation frequencies, types, and patterns between the L and S subtypes. Key findings include variations in mutation types, such as missense and frameshift mutations, and the recurrence percentages of colon cancer-related genes, highlighting the distinct mutational landscape of the 2 subtypes (Figure 5A and B).

In the L subtype, comprising 23 patient samples, all (100%) were found to harbor somatic mutations (Figure 5B). The oncoplot of the L subtype displayed the top 20 most frequently mutated genes, ranked by the total number of variants per gene, with the percentage reflecting the proportion of colon cancer samples with the genetic alteration relative to the total samples. Frequent mutations were observed in *APC* (83%), *TP53* (78%), *KRAS* (43%), *SYNE1* (35%), *MUC16* (30%), *TTN* (26%), and *KMT2B* (22%). These findings underscore the high mutation burden in this subtype. Similarly, in the S subtype, encompassing 16 patient samples, all (100%) were also found to have somatic mutations (Figure 5A). The most frequently mutated genes in this subtype included *APC* (75%), *TP53* (50%), *KRAS* (44%), *TTN* (44%), as well as *ADGRV1*, *AMER1*, *HMCN1*, *LRP1B*, *PIK3CA*, *SMAD4*, *SVEP1*, and *ZNF835*, each with a mutation frequency of 19%.

Across both subtypes, the top 3 most frequently mutated genes were *APC*, *TP53*, and *KRAS*. Other commonly mutated genes, *TTN*, *PIK3C*, and *MUC16*, were shared between the L and S subtypes.

### Tumor Cell Fractions in Cancer Subtypes

Recent studies have emphasized the significant role of the TME in the progression of colon cancer.^56,57^ A newly developed computational tool, known as Estimating the Proportions of Immune and Cancer Cells, was employed to assess the proportions of immune and cancer cells across the identified subtypes.^50^ The analysis revealed considerable variability in cell fractions between the 2 subtypes, particularly in the proportions of CD4 and CD8 T-cells. A statistically significant difference in immune cell expression was observed between the L and S subtypes (Kruskal-Wallis, *P* ⩽ .0035; Figure 3D, Supplemental S5 Figure). Notably, the late-stage S subtype showed a higher prevalence of immune cell fractions than the L subtype. The xCell tool shows similar expression patterns, with statistically significant differences in immune cell expression observed between the L and S subtypes of CD4 and CD8 T-cells (Kruskal-Wallis, *P* ⩽ .00092 and *P* ⩽ .00005, respectively; Supplemental S2 File). The *P*-values for each immune cell type (CD4 and CD8 T-cells) were adjusted for multiple testing using the FDR method. Because there was only 1 hypothesis test for each cell type, the adjusted *P*-values matched the original raw *P*-values. In the study, the decision to use multiple methods was aimed at enhancing the overall robustness of findings by identifying consistent patterns across different approaches. However, caution is warranted when interpreting results that diverge between the tools. This finding was corroborated in the independent GSE17538 dataset (Supplemental S2 File), where immune cell fractions were also significantly more abundant in the S subtype relative to the L subtype. These results suggest a distinct TME profile for each colon cancer subtype, with potential implications for tumor progression and immune response. Bulk deconvolution methods are sensitive to factors such as tumor purity and stromal composition, which can affect immune inference and introduce variability between datasets.^58^ Thus, our results should be viewed as overarching population trends that complement the more detailed mechanisms identified in recent studies.

In the Xena dataset, the EPIC-inferred CD8 T-cell fraction was inversely associated with the expression of oncogenes and TSGs, with the strongest single-gene correlations reaching a Spearman’s rho (ρ) of approximately −0.53. These correlations remained significant after multiple-testing corrections for many genes (Correlation Table.pdf). At the gene set level, higher CD8 fractions were associated with lower oncogene score expression (ρ = −0.34, *P* = .034) and lower TSG score expression (ρ = −0.37, *P* = .021). The CD4 fraction exhibited comparable negative trends, including a notable inverse correlation with the TSG score (ρ = −.35, *P* = .029) and a nearly significant association with the oncogene score (ρ = −.31, *P* = .053). Similarly, the CD8 and CD4 T-cell fractions were found to be inversely associated with the same oncogenes and TSGs in the GSE17538 dataset. Notably, stronger correlation results were obtained between CD8 and oncogenes (ρ = −.45, *P* = .002), CD8 and TSGs (ρ = −.58, *P* < .001), and between CD4 and oncogenes (ρ = −.48, *P* < .001) and CD4 and TSGs (ρ = −.55, *P* < .001). Because bulk expression may be confounded by tumor content, we repeated analyses adjusting for EPIC-estimated tumor fraction (otherCells). Subtype S remained strongly associated with reduced oncogene and TSG gene-set scores in purity-adjusted models (β ≈ −1.48 and β ≈ −1.54, both *P* < 10^−11^), while tumor purity was not a significant predictor in these models (Supplemental S2 Table). Additionally, all 16 RFE genes retained a significant subtype association after BH correction in models of the form *expression ~ subtype +* otherCells (Supplemental S3 Table).

## Discussion

Colon cancer is characterized by substantial intra-tumor heterogeneity and notable inter-patient variability, posing challenges to its understanding and treatment.^7,10^ While transcriptomic profiling has provided valuable insights into this heterogeneity, much of the existing genome and transcriptome-scale research has focused on primary tumors and matched normal tissues.^59-61^ Such approaches often fail to capture the dynamic, multi-stage progression of cancer from its early stages to late forms. This study explored the diverse progression of colon cancer by analyzing RNA-seq gene expression profiles across multiple stages of cancer development. This approach facilitated the identification of colon cancer subtypes and the exploration of genes with differential expression, mutational variations, TME, and enriched pathways linked to the subtypes.

Analyzing the gene expression profiles has significantly advanced our understanding of the variability in gene expression related to colon cancer progression. By uncovering a clear progression pattern from early to late stages of the disease, we identified 2 distinct colon cancer subtypes with distinct gene expression profiles (Figure 2A). If we consider the L subtype, the predominant form of colon cancer based on sample size, it appears that the S subtype demonstrates notable differences from the L subtype as the disease progresses. This distinction is strongly supported by the large number of DEGs identified between the 2 subtypes, totaling 1856. Interestingly, most of these DEGs, including both TSGs and oncogenes, were downregulated in the S subtype compared to the L subtype (Figures 2C, 3B and C). This downregulation may be ascribed to stress induced by the TME or gene silencing mechanisms potentially driven by mutations.

Furthermore, the 16-gene subset selected from the DEGs using the RFE method showed clear segregation between the 2 subtypes with 100% accuracy. This result highlights the efficiency of the RFE approach in rapidly identifying a minimal yet highly discriminative gene subset, further reinforcing the presence of substantial molecular heterogeneity between the 2 subtypes (Figure 2C and D). When applied to the independent GEO validation dataset, our in-house tracking cancer progression method successfully identified 2 distinct clusters, a large and a small cluster, supporting our findings. The ability to recover a similar separation of 2 clusters in an independent cohort suggests that the subtype signal is not cohort specific. However, results from the GEO dataset should be interpreted with caution, as several protein-coding genes, including some in the RFE gene subset, are unavailable. This limitation may have led to the misclassification of specific samples between clusters, potentially impacting downstream analyses if this dataset is used (Supplemental S1 Table). Hence, we acknowledge that the validation process is only partial. The boxplots show 6 RFE genes identified across both the Xena and GEO datasets (Figure 3A). Subtype L exhibited stable disease progression from early to late stages, with mean gene expression values remaining close to 0 throughout. In contrast, the S subtype showed significant downregulation of specific genes, indicating a notable increase in molecular activity (Figure 3A). Similarly, immune-related genes showed a stable expression around a mean value of 0 in L, while displaying upregulation in S, further supporting the notion of greater molecular dynamism in subtype S compared to L (Figure 3D).

A key finding of this study is the concurrent downregulation of both oncogenes and TSGs in S, which may be driven by likely random silencing mutations or regulatory mechanisms within the TME. Investigating the mutational landscape of the most frequently mutated genes, including known driver genes in colon cancer, revealed potential involvement of additional factors in their downregulation. For instance, *APC* is a well-known TSG,^62,63^ with mutation frequencies of 75% and 83% in S and L, respectively (Figure 5A and B). This difference could imply greater downregulation of *APC* in L due to its higher mutation frequency. However, this is not the case, as *APC* downregulation is more pronounced in the S subtype, indicating a possible role of TME in suppressing TSGs expression. Similarly, *KRAS* is a well-characterized oncogene in colon cancer,^64^ showing nearly identical mutation frequencies in both subtypes, yet its expression was more significantly downregulated in S than in L (Figure 5A and B). This further supports our assumption that additional factors, such as TME, contributed to the downregulation of oncogenes and TSGs observed in S.

The downregulation of some TSGs and oncogenes in subtype S is consistent with the observed miRNA profile. The differentially expressed miRNAs that target both TSGs and oncogenes were consistently upregulated, suggesting a pattern of gene inhibition.^65^ However, these links are based on externally curated functional MTIs and should therefore be interpreted as hypothesis-generating evidence rather than direct mechanistic confirmation in our cohort. The miRNA hsa-miR-106a-5p targeted the majority of TSGs and oncogenes. Previous studies have documented its role in colorectal cancer, linking it to tumor progression.^66,67^ This miRNA was not found to be significant in the L subtype, suggesting a different progression from the S subtype, confirming the validity of the method used to track cancer progression.^8^

Interestingly, a negative correlation was observed between the TME fractions of CD4 and CD8 T-cells and the DEGs, including TSGs and oncogenes. Both immune cell types manifested increased infiltration as the S subtype progressed, while the gene expression showed concurrent downregulation (Figure 3B-D). However, the observed relationships between the inferred immune cell fractions and expression of the TSGs and oncogenes should be interpreted as associations derived from bulk colorectal cancer transcriptomic data, and not as mechanistically proven or causal effects. The inverse relationship may reflect immune-linked transcriptional remodeling. However, bulk deconvolution does not resolve T-cell functional states, so mechanistic interpretation requires orthogonal validation.^66,67^ This phenomenon of immune-transcriptional disconnection has been documented in microsatellite-stable (MSS) colorectal cancers, specifically within the CMS2 and CMS3 classifications, where metabolic and epithelial processes prevail despite the presence of immune cells.^25,68^ In contrast, the L subtype showed increased expression of immune-related genes, along with more consistent regulation of tumor-associated genes. This suggests that the tumor is in a state that responds more effectively to immune activity. These findings are consistent with the immune-reactive phenotypes observed in MSI-high or CMS1 colorectal cancers, as reported by.^27,69^

A similar pattern of immune cell expression was observed in the GEO dataset analysis (Supplemental S3 Figure), further supporting our findings. Remarkably, CD4 and CD8 T-cells appeared to have a twofold effect on disease progression. While CD8 T-cells are generally known for their anti-tumor properties.^70^ Recent studies have highlighted their dual functionality, which varies depending on the TME and their state of exhaustion.^71,72^ Similarly, CD4 T-cells can exhibit both anti-tumorigenic and pro-tumorigenic roles, depending on their subset differentiation and functional state.^72,73^ This dual role contributed to a stabilized level of progression and, by extension, probably aggressiveness. Contrary to many previous reports,^73,74^ our findings suggest that TME does not necessarily exert a purely positive or negative influence on cancer aggressiveness. Instead, the presence of immune cell fractions with dual functions in the TME appears to possibly exert a balancing effect on colon cancer progression in the S subtype, as evidenced by the downregulation of both oncogenes and TSGs in this subtype. Consequently, if gene expression changes during disease progression from early to late stages are not accounted for, the 2 subtypes could be mistakenly regarded as a single subtype, underscoring the importance of the method for tracking cancer progression. However, the reported CD4^+^/CD8^+^ implications are descriptive and should not be viewed as mechanistic or causative without validation supported by raw data expression analyses and functional assays. Recent advances in single-cell and spatial transcriptomics have also improved understanding of the colorectal TME, showing that immune populations often have dual, context-dependent roles.^75,76^ However, our reliance on bulk transcriptomic data may mask distinct T-cell and stromal states, reflecting broader population signals and introducing variability due to sensitivity to tumor purity and composition.^58^ Thus, our findings should be viewed as complementing detailed mechanisms.

The 2 pathways identified in the KEGG pathway analysis, cell adhesion molecules and ECM receptor interaction, play crucial yet opposing roles in colon cancer (Figure 5C). The ECM receptor interaction pathway, through ECM remodeling, promotes a pro-tumorigenic environment that enhances disease progression.^55-78,79^ In contrast, the cell adhesion molecules pathway has been reported to support the anti-tumor response by facilitating the absorption of tumor antigens and activating T lymphocytes specific to the tumor location in colorectal cancer.^80^ The downregulation of both pathways during disease progression results in an altered effect that maintains a consistent level of aggressiveness. This further explains our finding regarding the balancing effect of the TME on colon cancer progression in the S subtype. Moreover, a recent report by^81^ also identified a strong correlation between immune cell infiltration and the ECM receptor signatures. These insights provide a new foundation for the future design of anticancer drugs, focusing on the unique molecular characteristics of the identified subtypes and the TME. It is interesting to note that the enriched pathways identified in Reactome, collagen chain trimerization and the RHOA GTPase cycle, exhibit similar biological functions to those found in the KEGG pathways. Specifically, collagen chain trimerization plays a comparable role to cell adhesion molecules within the extracellular matrix, influencing both the structure and function.^81,82^ Furthermore, ECM interactions significantly regulate RhoA activity,^83^ further supporting our findings. This convergence of pathways highlights the interconnectedness of these biological processes and underscores the relevance of our research.

## Conclusion

Our study demonstrates a complex relationship between TME and gene expression that helps explain the molecular heterogeneity of novel colon cancer subtypes. The simultaneous downregulation of both TSGs and oncogenes in the S subtype suggests that factors beyond mutational burden, such as TME regulation mechanisms, contribute to this downregulation. In addition, the regulation of cancer driver genes is negatively correlated with CD4 and CD8 T-cells, suggesting a likely dual role of immune regulation in controlling S subtype progression by modulating TSGs and oncogenes that play opposing roles in cancer. Without studying tumor progression across stages, distinguishing the subtypes would be difficult and easily overlooked. The strong findings here enhance our understanding of the genetic characteristics and molecular mechanisms involved in the heterogeneity and progression of colon cancer. This knowledge improves colon cancer prognosis and advances the development of precise, targeted therapies.

## Supplemental Material

sj-docx-1-cix-10.1177_11769351261431245 – Supplemental material for In Silico Analysis of the Dual Role of Tumor Microenvironment on Colon Cancer Subtypes Supplemental material, sj-docx-1-cix-10.1177_11769351261431245 for In Silico Analysis of the Dual Role of Tumor Microenvironment on Colon Cancer Subtypes by Christianah Kehinde, Michelle Livesey, Yeuko Manganyi and Hocine Bendou in Cancer Informatics

sj-docx-2-cix-10.1177_11769351261431245 – Supplemental material for In Silico Analysis of the Dual Role of Tumor Microenvironment on Colon Cancer Subtypes Supplemental material, sj-docx-2-cix-10.1177_11769351261431245 for In Silico Analysis of the Dual Role of Tumor Microenvironment on Colon Cancer Subtypes by Christianah Kehinde, Michelle Livesey, Yeuko Manganyi and Hocine Bendou in Cancer Informatics

## References

[1] Sawicki T Ruszkowska M Danielewicz A Niedźwiedzka E Arłukowicz T Przybyłowicz KE. A review of colorectal cancer in terms of epidemiology, risk factors, development, symptoms and diagnosis. Cancers. 2021;13(9):2025. 33922197 10.3390/cancers13092025 PMC8122718

[2] Ashique S Bhowmick M Pal R , et al. Multi drug resistance in Colorectal Cancer- approaches to overcome, advancements and future success. Adv Cancer Biol Metastasis. 2024;10:100114.

[3] Sung H Ferlay J Siegel RL , et al. Global Cancer Statistics 2020: GLOBOCAN estimates of incidence and mortality worldwide for 36 cancers in 185 countries. CA Cancer J Clin. 2021;71(3):209-249. 33538338 10.3322/caac.21660

[4] Ferlay J Colombet M Soerjomataram I , et al. Cancer statistics for the year 2020: an overview. Int J Cancer. 2021;149(4):778-789. 10.1002/ijc.33588 33818764

[5] Xi Y Xu P. Global colorectal cancer burden in 2020 and projections to 2040. Transl Oncol. 2021;14(10):101174. 10.1016/j.tranon.2021.101174 PMC8273208 34243011

[6] Testa U Pelosi E Castelli G. Colorectal cancer: genetic abnormalities, tumor progression, tumor heterogeneity, clonal evolution and tumor-initiating cells. Med Sci. 2018;6(2):31. 10.3390/medsci6020031 PMC6024750 29652830

[7] Schmitt M Greten FR. The inflammatory pathogenesis of colorectal cancer. Nat Rev Immunol. 2021;21(10):653-667. 33911231 10.1038/s41577-021-00534-x

[8] Dienstmann R Mason MJ Sinicrope FA , et al. Prediction of overall survival in stage II and III colon cancer beyond TNM system: a retrospective, pooled biomarker study. Ann Oncol. 2017;28(5):1023-1031. 28453697 10.1093/annonc/mdx052 PMC5406760

[9] Sagaert X Vanstapel A Verbeek S. Tumor heterogeneity in colorectal cancer: what do we know so far? Pathobiology. 2018;85(1-2):72-84. 29414818 10.1159/000486721

[10] Seligmann JF. Colorectal cancer staging-time for a re-think on TNM? Br J Surg. 2025;112(3):znaf047. 10.1093/bjs/znaf047 40067086

[11] Buikhuisen JY Torang A Medema JP. Exploring and modelling colon cancer inter-tumour heterogeneity: opportunities and challenges. Oncogenesis. 2020;9(7):66. 32647253 10.1038/s41389-020-00250-6 PMC7347540

[12] Wen R Zhou L Peng Z , et al. Single-cell sequencing technology in colorectal cancer: a new technology to disclose the tumor heterogeneity and target precise treatment. Front Immunol. 2023;14:1175343. 10.3389/fimmu.2023.1175343 PMC10225552 37256123

[bibr13-11769351261431245] Sobral D Martins M Kaplan S , et al. Genetic and microenvironmental intra-tumor heterogeneity impacts colorectal cancer evolution and metastatic development. Commun Biol. 2022;5(1):937. 36085309 10.1038/s42003-022-03884-x PMC9463147

[bibr14-11769351261431245] Li Q Geng S Luo H , et al. Signaling pathways involved in colorectal cancer: pathogenesis and targeted therapy. Signal Transduct Target Ther. 2024;9(1):266-348. 39370455 10.1038/s41392-024-01953-7 PMC11456611

[15] Al-Joufi FA Setia A Salem-Bekhit MM , et al. Molecular pathogenesis of colorectal cancer with an emphasis on recent advances in biomarkers, as well as nanotechnology-based diagnostic and therapeutic approaches. Nanomater. 2022;12(1):169. 10.3390/nano12010169 PMC8746463 35010119

[16] Pernot S Evrard S Khatib AM. The Give-and-Take interaction between the tumor microenvironment and immune cells regulating tumor progression and repression. Front Immunol. 2022;13:850856. 10.3389/fimmu.2022.850856 PMC9043524 35493456

[17] Goyette MA Lipsyc-Sharf M Polyak K. Clinical and translational relevance of intratumor heterogeneity. Trends Cancer. 2023;9(9):726-737. 37248149 10.1016/j.trecan.2023.05.001 PMC10524913

[18] Punt CJ Koopman M Vermeulen L. From tumour heterogeneity to advances in precision treatment of colorectal cancer. Nat Rev Clin Oncol. 2017;14(4):235-246. 27922044 10.1038/nrclinonc.2016.171

[19] Li A Li Q Wang C , et al. Constructing a prognostic model for colon cancer: insights from immunity-related genes. BMC Cancer. 2024;24(1):758. 38914961 10.1186/s12885-024-12507-z PMC11197172

[bibr20-11769351261431245] Nunes L Li F Wu M , et al. Prognostic genome and transcriptome signatures in colorectal cancers. Nature. 2024;633(8028):137-146. 39112715 10.1038/s41586-024-07769-3 PMC11374687

[21] Yazdani A Lenz HJ Pillonetto G , et al. Gene signatures derived from transcriptomic-causal networks stratify colorectal cancer patients for effective targeted therapy. Commun Med. 2025;5(1):9. 39779996 10.1038/s43856-024-00728-z PMC11711454

[22] Peña-Romero AC Orenes-Piñero E. Dual effect of immune cells within tumour microenvironment: pro- and anti-tumour effects and their triggers. Cancers. 2022;14(7):1681. 35406451 10.3390/cancers14071681 PMC8996887

[23] Guo Y Shang X Li Z. Identification of cancer subtypes by integrating multiple types of transcriptomics data with deep learning in breast cancer. Neurocomputing. 2019;324:20-30.

[24] Lu J Chen Q. Transcriptome-based identification of molecular markers related to the development and prognosis of colon cancer. Nucleosides Nucleotides Nucleic Acids. 2021;40(11):1114-1124. 34519615 10.1080/15257770.2021.1975298

[25] Mokhtari K Peymani M Rashidi M , et al. Colon cancer transcriptome. Prog Biophys Mol Biol. 2023;180-181:49-82. 37059270 10.1016/j.pbiomolbio.2023.04.002

[26] Kim R Schell MJ Teer JK Greenawalt DM Yang M Yeatman TJ. Co-evolution of somatic variation in primary and metastatic colorectal cancer may expand biopsy indications in the molecular era. PLoS One. 2015;10(5):e0126670. 10.1371/journal.pone.0126670 PMC4431733 25974029

[27] Guinney J Dienstmann R Wang X , et al. The consensus molecular subtypes of Colorectal Cancer. Nat Med. 2015;21(11):1350-1356. 26457759 10.1038/nm.3967 PMC4636487

[28] Fontana E Eason K Cervantes A Salazar R Sadanandam A. Context matters-consensus molecular subtypes of colorectal cancer as biomarkers for clinical trials. Ann Oncol. 2019;30(4):520-527. 30796810 10.1093/annonc/mdz052 PMC6503627

[29] Pfeiffer P Qvortrup C. Does the consensus molecular subtypes classification add to selection of precision medicine in patients with metastatic colorectal cancer? Dig Med Res. 2020;3(0):20-20.

[30] Livesey M Eshibona N Bendou H. Assessment of the progression of kidney renal clear cell carcinoma using transcriptional profiles revealed new cancer subtypes with variable prognosis. Front Genet. 2023;14:1291043. 10.3389/fgene.2023.1291043 PMC10704507 38075696

[31] Livesey M Rossouw SC Blignaut R Christoffels A Bendou H. Transforming RNA-Seq gene expression to track cancer progression in the multi-stage early to advanced-stage cancer development. PLoS One. 2023;18(4):e0284458. 10.1371/journal.pone.0284458 PMC10124877 37093793

[32] Vilar E Tabernero J. Molecular dissection of microsatellite instable colorectal cancer. Cancer Discov. 2013;3(5):502-511. 23454900 10.1158/2159-8290.CD-12-0471 PMC3651752

[33] Weisenberger DJ Siegmund KD Campan M , et al. CpG island methylator phenotype underlies sporadic microsatellite instability and is tightly associated with BRAF mutation in colorectal cancer. Nat Genet. 2006;38(7):787-793. 16804544 10.1038/ng1834

[34] Goldman MJ Craft B Hastie M , et al. Visualizing and interpreting cancer genomics data via the Xena platform. Nat Biotechnol. 2020;38(6):675-678. 32444850 10.1038/s41587-020-0546-8 PMC7386072

[35] Cunningham F Allen JE Allen J , et al. Ensembl 2022. Nucleic Acids Res. 2022;50(D1):D988-D995. 10.1093/nar/gkab1049 PMC8728283 34791404

[36] Smedley D Haider S Durinck S , et al. The BioMart community portal: an innovative alternative to large, centralized data repositories. Nucleic Acids Res. 2015;43(W1):W589-W598. 10.1093/nar/gkv350 PMC4489294 25897122

[37] Tokuda EK Comin CH Costa LDF . Revisiting agglomerative clustering. Phys Stat Mech Its Appl. 2022;585:126433.

[38] Dinh DT Fujinami T Huynh VN. Estimating the optimal number of clusters in categorical data clustering by silhouette coefficient. In: Chen J Huynh VN Nguyen GN Tang X , eds. Knowledge and Systems Sciences. Springer; 2019;1-17.

[39] Ritchie ME Phipson B Wu D , et al. Limma powers differential expression analyses for RNA-sequencing and microarray studies. Nucleic Acids Res. 2015;43(7):e47. 10.1093/nar/gkv007 PMC4402510 25605792

[40] Law CW Alhamdoosh M Su S Smyth GK Ritchie ME. RNA-seq analysis is easy as 1-2-3 with limma, glimma and edgeR. F1000Res. 2016;5:1408. 10.12688/f1000research.9005.1 PMC4937821 27441086

[41] Trory JS Munkacsi A Śledź KM , et al. Chemical degradation of BTK/TEC as a novel approach to inhibit platelet function. Blood Adv. 2023;7(9):1692-1696. 36342848 10.1182/bloodadvances.2022008466 PMC10182296

[42] Mahin KF Robiuddin M Islam M Ashraf S Yeasmin F Shatabda S. PanClassif: Improving pan cancer classification of single cell RNA-seq gene expression data using machine learning. Genomics. 2022;114(2):110264. 10.1016/j.ygeno.2022.01.001 34998929

[43] Talb J Takam Kamga P Mayenga M , et al. Gene expression profile of high PD-L1 non-small cell lung cancers refractory to pembrolizumab. Cancer Immunol Immunother. 2022;71(11):2791-2799. 35435450 10.1007/s00262-022-03206-4 PMC10992407

[44] Song YJ Peng B zheng L. Expression pattern of osteoarthritis is tied to the matrix microenvironmen. Osteoarthr Cartil. 2024;32:S465-S466.

[45] Wu T Hu E Xu S , et al. clusterProfiler 4.0: A universal enrichment tool for interpreting omics data. Innovation. 2021;2(3):100141. 10.1016/j.xinn.2021.100141 PMC8454663 34557778

[46] Gene Ontology Consortium. The Gene Ontology resource: enriching a GOld mine. Nucleic Acids Res. 2021;49(D1):D325-D334. 10.1093/nar/gkaa1113 PMC7779012 33290552

[47] Jassal B Matthews L Viteri G , et al. The reactome pathway knowledgebase. Nucleic Acids Res. 2020;48(D1):D498-D503. 10.1093/nar/gkz1031 PMC7145712 31691815

[48] Mayakonda A Lin DC Assenov Y Plass C Koeffler HP. Maftools: efficient and comprehensive analysis of somatic variants in cancer. Genome Res. 2018;28(11):1747-1756. 30341162 10.1101/gr.239244.118 PMC6211645

[49] Racle J de Jonge K Baumgaertner P Speiser DE Gfeller D. Simultaneous enumeration of cancer and immune cell types from bulk tumor gene expression data. eLife. 2017;6:e26476. 10.7554/eLife.26476 PMC5718706 29130882

[50] Racle J Gfeller D. EPIC: A tool to estimate the proportions of different cell types from bulk gene expression data. In: Boegel S , ed. Bioinformatics for Cancer Immunotherapy: Methods and Protocols. Springer US; 2020;233-248. 10.1007/978-1-0716-0327-7_17 32124324

[bibr51-11769351261431245] Aran D Hu Z Butte AJ. xCell: digitally portraying the tissue cellular heterogeneity landscape. Genome Biol. 2017;18(1):220. 29141660 10.1186/s13059-017-1349-1 PMC5688663

[52] Angel A Naom L Nabet-Levy S Aran D. xCell 2.0: robust algorithm for cell type proportion estimation predicts response to immune checkpoint blockade. Bioinformatics. Preprint posted online September 10, 2024 2025;26:335. doi:10.1186/s13059-025-03784-3 PMC12492622 41044684

[53] Clough E Barrett T Wilhite SE , et al. NCBI GEO: archive for gene expression and epigenomics data sets: 23-year update. Nucleic Acids Res. 2024;52(D1):D138-D144. 10.1093/nar/gkad965 PMC10767856 37933855

[54] Sondka Z Dhir NB Carvalho-Silva D , et al. COSMIC: a curated database of somatic variants and clinical data for cancer. Nucleic Acids Res. 2024;52(D1):D1210-D1217. 10.1093/nar/gkad986 PMC10767972 38183204

[55] Li ZL Wang ZJ Wei GH Yang Y Wang XW. Changes in extracellular matrix in different stages of colorectal cancer and their effects on proliferation of cancer cells. World J Gastrointest Oncol. 2020;12(3):267-275. 32206177 10.4251/wjgo.v12.i3.267 PMC7081112

[56] Cheruku S Rao V Pandey R Rao Chamallamudi M Velayutham R Kumar N. Tumor-associated macrophages employ immunoediting mechanisms in colorectal tumor progression: Current research in Macrophage repolarization immunotherapy. Int Immunopharmacol. 2023;116:109569. 10.1016/j.intimp.2022.109569 36773572

[57] Nicolini A Ferrari P. Involvement of tumor immune microenvironment metabolic reprogramming in colorectal cancer progression, immune escape, and response to immunotherapy. Front Immunol. 2024;15:1353787. 10.3389/fimmu.2024.1353787 PMC11306065 39119332

[58] Lu S Yang J Yan L , et al. Transcriptome size matters for single-cell RNA-seq normalization and bulk deconvolution. Nat Commun. 2025;16(1):1246. 39893178 10.1038/s41467-025-56623-1 PMC11787294

[59] Vignot S Lefebvre C Frampton GM , et al. Comparative analysis of primary tumour and matched metastases in colorectal cancer patients: Evaluation of concordance between genomic and transcriptional profiles. Eur J Cancer. 2015;51(7):791-799. 25797355 10.1016/j.ejca.2015.02.012

[60] Yi H Liao ZW Chen JJ , et al. Genome variation in colorectal cancer patient with liver metastasis measured by whole-exome sequencing. J Gastrointest Oncol. 2021;12(2):507-515. 34012644 10.21037/jgo-21-9 PMC8107593

[61] Pinkney HR Ross CR Hodgson TO Pattison ST Diermeier SD. Discovery of prognostic lncRNAs in colorectal cancer using spatial transcriptomics. Npj Precis Oncol. 2024;8(1):230. 39390212 10.1038/s41698-024-00728-1 PMC11467462

[62] Hankey W Frankel WL Groden J. Functions of the APC tumor suppressor protein dependent and independent of canonical WNT signaling: Implications for therapeutic targeting. Cancer Metastasis Rev. 2018;37(1):159-172. 29318445 10.1007/s10555-017-9725-6 PMC5803335

[bibr63-11769351261431245] Schatoff EM Goswami S Zafra MP , et al. Distinct colorectal cancer-associated APC mutations dictate response to tankyrase inhibition. Cancer Discov. 2019;9(10):1358-1371. 31337618 10.1158/2159-8290.CD-19-0289 PMC6774804

[64] Dienstmann R Connor K Byrne AT , et al. Precision Therapy in RAS Mutant Colorectal Cancer. Gastroenterology. 2020;158(4):806-811. 31972237 10.1053/j.gastro.2019.12.051

[65] Otmani K Lewalle P. Tumor suppressor miRNA in cancer cells and the tumor microenvironment: Mechanism of deregulation and clinical implications. Front Oncol. 2021;11:708765. 10.3389/fonc.2021.708765 PMC8554338 34722255

[66] Liang Y Li J Yuan Y , et al. Exosomal miR-106a-5p from highly metastatic colorectal cancer cells drives liver metastasis by inducing macrophage M2 polarization in the tumor microenvironment. J Exp Clin Cancer Res CR. 2024;43:281. 39385295 10.1186/s13046-024-03204-7 PMC11462797

[67] Peng Q Shen Y Zhao P Cheng M Zhu Y Xu B. Biomarker roles identification of miR-106 family for predicting the risk and poor survival of colorectal cancer. BMC Cancer. 2020;20:506. 32493303 10.1186/s12885-020-06863-9 PMC7268235

[68] Olguín JE Medina-Andrade I Rodríguez T Rodríguez-Sosa M Terrazas LI. Relevance of regulatory T cells during colorectal cancer development. Cancers. 2020;12(7):1888. 32674255 10.3390/cancers12071888 PMC7409056

[69] Zhang M Wang HZ Peng RY Xu F Wang F Zhao Q. Metabolism-associated molecular classification of Colorectal Cancer. Front Oncol. 2020;10:602498. 10.3389/fonc.2020.602498 PMC7746835 33344254

[70] Raskov H Orhan A Christensen JP Gögenur I. Cytotoxic CD8+ T cells in cancer and cancer immunotherapy. Br J Cancer. 2021;124(2):359-367. 32929195 10.1038/s41416-020-01048-4 PMC7853123

[71] Huang Y Jia A Wang Y Liu G. CD8+ T cell exhaustion in anti-tumour immunity: the new insights for cancer immunotherapy. Immunology. 2023;168(1):30-48. 36190809 10.1111/imm.13588

[72] Zheng Z Wieder T Mauerer B Schäfer L Kesselring R Braumüller H. T cells in colorectal cancer: Unravelling the function of different T cell subsets in the tumor microenvironment. Int J Mol Sci. 2023;24(14):11673. 10.3390/ijms241411673 PMC10380781 37511431

[73] Toor SM Murshed K Al-Dhaheri M Khawar M Abu Nada M Elkord E. Immune checkpoints in circulating and tumor-infiltrating CD4+ T cell subsets in Colorectal Cancer Patients. Front Immunol. 2019;10:2936. 31921188 10.3389/fimmu.2019.02936 PMC6928042

[74] Waldner M Schimanski CC Neurath MF. Colon cancer and the immune system: the role of tumor invading T cells. World J Gastroenterol. 2006;12(45):7233-7238. 17143936 10.3748/wjg.v12.i45.7233 PMC4087478

[75] Xiao J Yu X Meng F , et al. Integrating spatial and single-cell transcriptomics reveals tumor heterogeneity and intercellular networks in colorectal cancer. Cell Death Discov. 2024;15(5):326. 10.1038/s41419-024-06598-6 PMC11087651 38729966

[76] Ozato Y Kojima Y Kobayashi Y , et al. Spatial and single-cell transcriptomics decipher the cellular environment containing HLA-G+ cancer cells and SPP1+ macrophages in colorectal cancer. Cell Rep. 2023;42(1):111929. 10.1016/j.celrep.2022.111929 36656712

[77] Terzi A Maqoud F Guido D , et al. Ion Channel-Extracellular Matrix Interplay in Colorectal Cancer: A Network-Based Approach to Tumor Microenvironment Remodeling. Int J Mol Sci. 2025;26(11):5147. 40507957 10.3390/ijms26115147 PMC12154350

[78] Chai R Su Z Zhao Y Liang W. Extracellular matrix-based gene signature for predicting prognosis in colon cancer and immune microenvironment. Transl Cancer Res. 2023;12(2):321-339. 36915600 10.21037/tcr-22-2036 PMC10007896

[79] Nersisyan S Novosad V Engibaryan N Ushkaryov Y Nikulin S Tonevitsky A. ECM-Receptor regulatory network and its prognostic role in colorectal cancer. Front Genet. 2021;12:782699. 10.3389/fgene.2021.782699 PMC8685507 34938324

[80] Qiu Z Wang Y Zhang Z , et al. Roles of intercellular cell adhesion molecule-1 (ICAM-1) in colorectal cancer: expression, functions, prognosis, tumorigenesis, polymorphisms and therapeutic implications. Front Oncol. 2022;12:1052672. 10.3389/fonc.2022.1052672 PMC9728583 36505809

[81] Bourgot I Primac I Louis T Noël A Maquoi E. Reciprocal interplay between fibrillar collagens and collagen-binding integrins: Implications in cancer progression and metastasis. Front Oncol. 2020;10:1488. 33014790 10.3389/fonc.2020.01488 PMC7461916

[82] Elango J Hou C Bao B Wang S Maté Sánchez de Val JE Wenhui W. The molecular interaction of collagen with cell receptors for biological function. Polymers. 2022;14(5):876. 35267698 10.3390/polym14050876 PMC8912536

[83] Nayak RC Chang KH Vaitinadin NS Cancelas JA. Rho GTPases control specific cytoskeleton-dependent functions of hematopoietic stem cells. Immunol Rev. 2013;256(1):255-268. 24117826 10.1111/imr.12119 PMC3830525

