# Supplementary material for: In Silico Analysis of the Dual Role of Tumor Microenvironment on Colon Cancer Subtypes
Source: Cancer Inform. 2026 Mar 26;25:11769351261431245. doi: 10.1177/11769351261431245 (PMC13033067; doi:10.1177/11769351261431245)
Supplement: sj-docx-1-cix-10.1177_11769351261431245 – Supplemental material for In Silico Analysis of the Dual Role of Tumor Microenvironment on Colon Cancer Subtypes [file sj-docx-1-cix-10.1177_11769351261431245.docx]

S1 File: Validation of the XENA dataset findings using an independent dataset GSE17538 to support the robustness of the results.

**
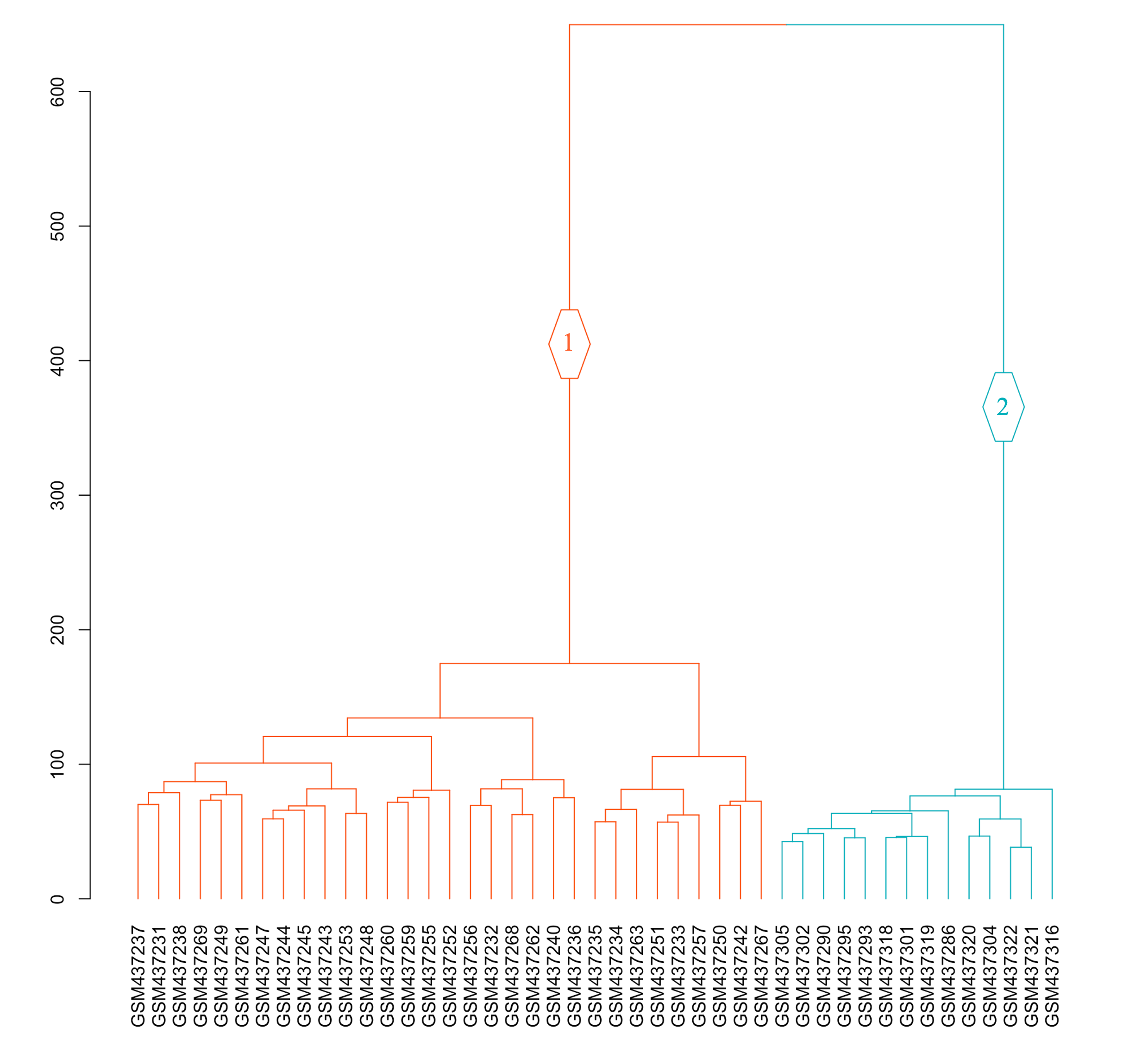
**

**S1 Fig:** Hierarchical clustering dendrogram of colon cancer samples in the GEO dataset. The normalized gene expression of the 45 colon cancer samples was subjected to hierarchical clustering analysis and revealed two clusters: L (orange branches) with 31 samples and S (blue branches) with 14 samples.


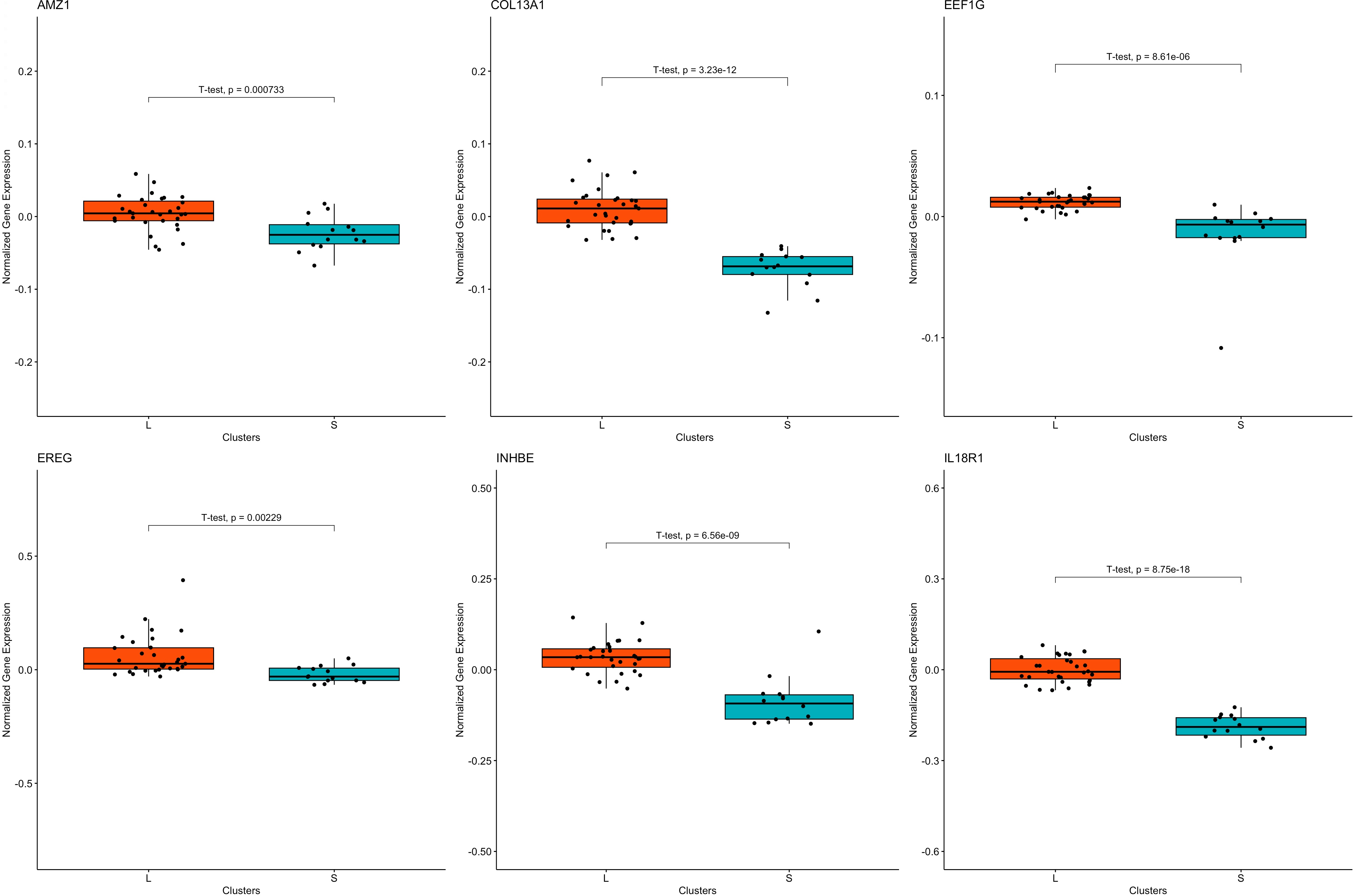


**S2 Fig**: Boxplots of six of the RFE gene subset from the GEO dataset. Boxplots depict the normalized gene expression profiles of six genes (*AMZ1*, *COL13A1*, *EEF1G*, *EREG*, *IL18R1* and *INHBE*) in colon cancer samples classified into the L and S clusters. Also, these genes were showing the same gene expression pattern in the Xena dataset.


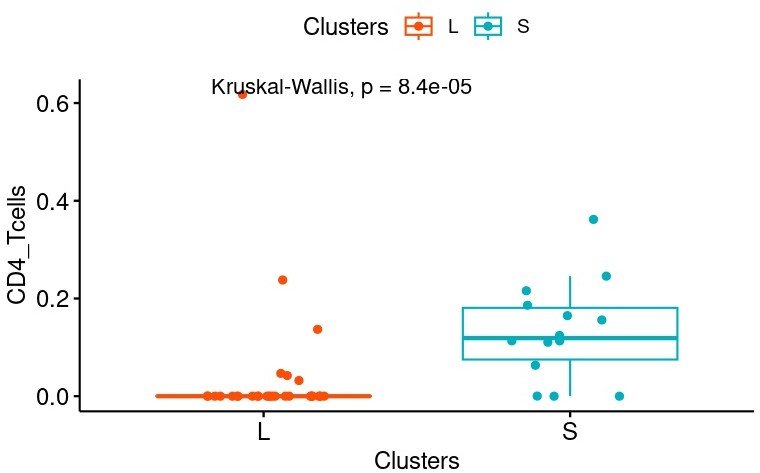

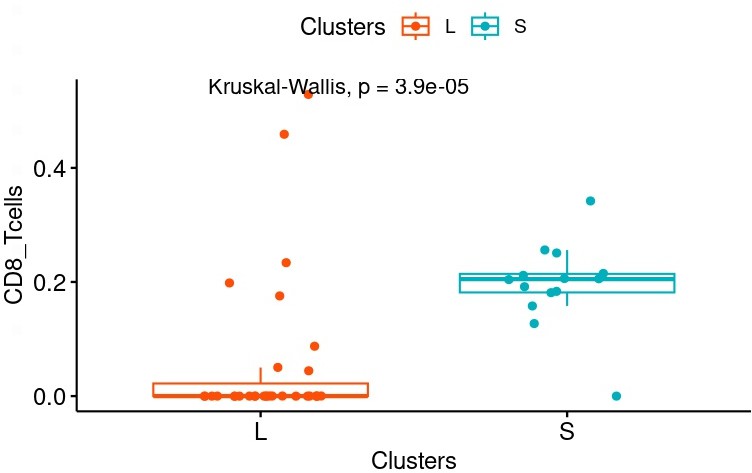


**S3 Fig**: Boxplots of immune cell fractions in colon cancer clusters from the GEO dataset. Boxplots illustrate the distribution of immune cell fractions for CD4 and CD8 T-cells across the L and S subtypes in the GEO dataset. A comparable pattern of immune cell expression was observed in the analysis of the Xena dataset.


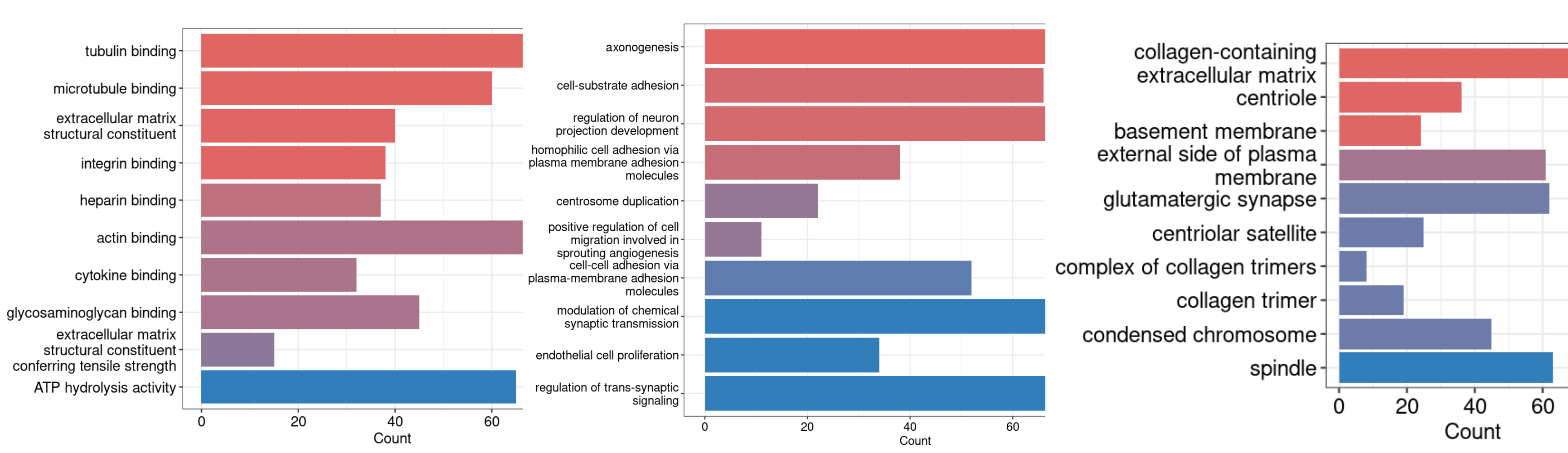


**S4 Fig:** GO pathway enrichment analysis. GO-(CC, BP and MF) enrichment analysis of the 1,855 DEGs was performed using the clusterProfiler R package, identified top enriched pathways including collagen-containing extracellular matrix centriole, extracellular matrix structural constituent, and cell substrate adhesion.


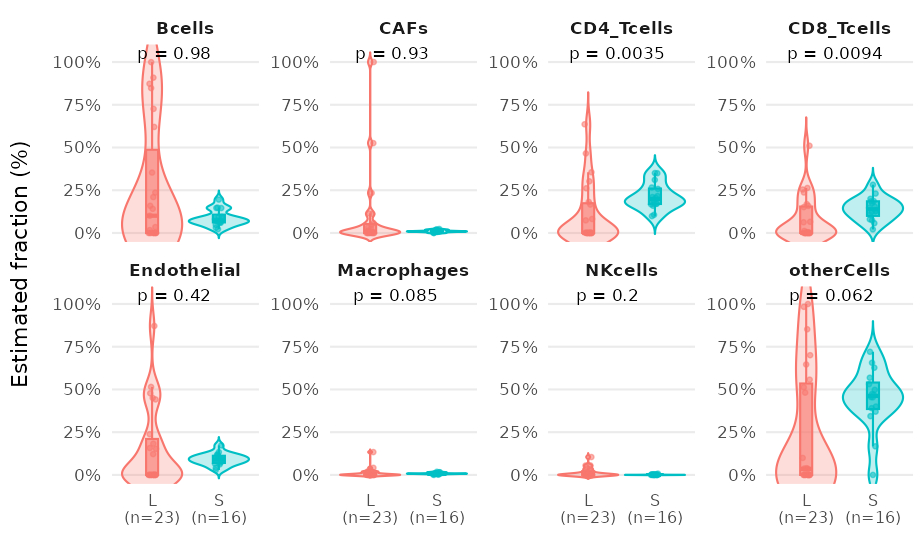
**S5 Fig:** EPIC immune/stromal deconvolution by subtype. Boxplots show EPIC-estimated cell-type fractions (%) across subtypes L (n=23) and S (n=16). P-values (Wilcoxon rank-sum, two-sided) are indicated above each panel. CD4 and CD8 T-cell fractions are significantly higher in subtype S (CD4: p=0.0035; CD8: p=0.0094), while differences for B cells, CAFs, endothelial cells, macrophages, NK cells, and the aggregated “otherCells” component are not significant at α=0.05.
