# Supplementary material for: In Silico Analysis of the Dual Role of Tumor Microenvironment on Colon Cancer Subtypes
Source: Cancer Inform. 2026 Mar 26;25:11769351261431245. doi: 10.1177/11769351261431245 (PMC13033067; doi:10.1177/11769351261431245)
Supplement: sj-docx-2-cix-10.1177_11769351261431245 – Supplemental material for In Silico Analysis of the Dual Role of Tumor Microenvironment on Colon Cancer Subtypes [file sj-docx-2-cix-10.1177_11769351261431245.docx]

**S1 Table: RFE selected 16-gene subset with the highest performance score of 0.977.** The first six genes listed were found in both Xena and the independent GEO validation dataset.

| **Ensembl stable ID** | **Gene name** | **Gene Expression Pattern** |
| --- | --- | --- |
| ENSG00000174945 | *AMZ1** | Downregulation |
| ENSG00000197467 | *COL13A1** | Downregulation |
| ENSG00000254772 | *EEF1G** | Downregulation |
| ENSG00000124882 | *EREG** | Downregulation |
| ENSG00000115604 | *IL18R1** | Downregulation |
| ENSG00000139269 | *INHBE** | Downregulation |
| ENSG00000099977 | *DDT* | Downregulation |
| ENSG00000129226 | *CD68* | Downregulation |
| ENSG00000160201 | *U2AF1* | Downregulation |
| ENSG00000160469 | *BRSK1* | Downregulation |
| ENSG00000204967 | *PCDHA4* | Downregulation |
| ENSG00000258102 | *MAP1LC3B2* | Downregulation |
| ENSG00000274512 | *TBC1D3L* | Downregulation |
| ENSG00000259171 | - | Downregulation |
| ENSG00000262304 | - | Downregulation |
| ENSG00000271741 | - | Downregulation |

* Genes found in both Xena and the independent GEO validation dataset.

-No gene name available using Biomart (GRCh38.p14, Ensembl release 113, April 2024)

**S2 Table: Purity-adjusted linear models for oncogene and tumor-suppressor gene-set scores.** Oncogene and TSG gene-set scores were modeled as a function of subtype (cluster S vs reference cluster L) while adjusting for EPIC-estimated tumor cell fraction. The table reports regression coefficients (estimate), standard errors, t-statistics, and p-values.

| term | estimate | std.error | statistic | p.value | score |
| --- | --- | --- | --- | --- | --- |
| (Intercept) | 0.5463412 | 0.10922506 | 5.00197649 | 1.49E-05 | onc_score |
| clusterS | -1.4831383 | 0.14802469 | -10.019533 | 5.90E-12 | onc_score |
| otherCells | 0.18539333 | 0.23674817 | 0.78308244 | 0.43870012 | onc_score |
| (Intercept) | 0.60433515 | 0.10480592 | 5.76623088 | 1.43E-06 | tsg_score |
| clusterS | -1.543358 | 0.14203576 | -10.865982 | 6.45E-13 | tsg_score |
| otherCells | 0.08605531 | 0.22716957 | 0.37881532 | 0.70705005 | tsg_score |

**S3 Table. Purity-adjusted linear models for the 16 RFE-selected genes.** For each of the 16 genes selected by recursive feature elimination (RFE), we fit a linear model of the form *expression ~ subtype (S vs L) + EPIC-estimated tumor fraction* to assess whether subtype-associated differential expression persists after accounting for tumor cellularity. The table reports regression coefficients (estimate), standard errors, test statistics, nominal p-values, and Benjamini-Hochberg-adjusted q-values for both subtype and otherCells terms.

| Gene | beta_subtype_S | p_subtype_S | beta_tumorCells | p_tumorCells | q_subtype_S | q_tumorCells |
| --- | --- | --- | --- | --- | --- | --- |
| ENSG00000259171 | -2.5732462 | 9.39E-16 | -0.092163 | 0.76245568 | 1.50E-14 | 0.96322514 |
| ENSG00000129226 | -2.4159792 | 5.79E-15 | 0.03290339 | 0.91381338 | 4.63E-14 | 0.96322514 |
| ENSG00000271741 | -1.7674981 | 5.26E-13 | 0.28010519 | 0.28530958 | 2.39E-12 | 0.91461002 |
| ENSG00000104880 | -1.5037481 | 5.97E-13 | -0.0934364 | 0.67458147 | 2.39E-12 | 0.96322514 |
| ENSG00000154736 | -1.6509847 | 3.67E-12 | -0.0534241 | 0.83769786 | 1.17E-11 | 0.96322514 |
| ENSG00000254772 | -2.2022392 | 6.42E-12 | -0.0254221 | 0.94293021 | 1.71E-11 | 0.96322514 |
| ENSG00000161955 | -1.714599 | 9.45E-12 | 0.32776994 | 0.24709119 | 2.13E-11 | 0.91461002 |
| ENSG00000249709 | -1.5423072 | 1.07E-11 | -0.0991134 | 0.69612063 | 2.13E-11 | 0.96322514 |
| ENSG00000161654 | -1.9524799 | 1.22E-11 | 0.01486976 | 0.96322514 | 2.16E-11 | 0.96322514 |
| ENSG00000274512 | -2.5594788 | 2.02E-10 | 0.24988244 | 0.59710804 | 3.22E-10 | 0.96322514 |
| ENSG00000080573 | -1.8119818 | 6.42E-10 | 0.37689487 | 0.28581563 | 9.34E-10 | 0.91461002 |
| ENSG00000259024 | -1.5254651 | 1.26E-09 | -0.1241079 | 0.6828033 | 1.68E-09 | 0.96322514 |
| ENSG00000238083 | -1.6484996 | 1.41E-09 | 0.11392983 | 0.72969849 | 1.74E-09 | 0.96322514 |
| ENSG00000136720 | -1.427811 | 1.58E-09 | -0.5607392 | 0.05661758 | 1.80E-09 | 0.90588124 |
| ENSG00000270757 | -1.6468609 | 4.09E-09 | 0.47130422 | 0.1769304 | 4.36E-09 | 0.91461002 |
| ENSG00000174945 | -1.8447765 | 4.26E-07 | 0.23998253 | 0.61942362 | 4.26E-07 | 0.96322514 |
